# Using Propensity Scores for Causal Inference: Pitfalls and Tips

**DOI:** 10.2188/jea.JE20210145

**Published:** 2021-08-05

**Authors:** Koichiro Shiba, Takuya Kawahara

**Affiliations:** 1 Department of Epidemiology, Harvard T.H. Chan School of Public Health, Boston, MA, USA; 2 Department of Social and Behavioral Sciences, Harvard T.H. Chan School of Public Health, Boston, MA, USA; 3 Clinical Research Promotion Center, The University of Tokyo Hospital, Tokyo, Japan

**Keywords:** propensity score, matching, inverse probability weighting, target population

## Abstract

Methods based on propensity score (PS) have become increasingly popular as a tool for causal inference. A better understanding of the relative advantages and disadvantages of the alternative analytic approaches can contribute to the optimal choice and use of a specific PS method over other methods. In this article, we provide an accessible overview of causal inference from observational data and two major PS-based methods (matching and inverse probability weighting), focusing on the underlying assumptions and decision-making processes. We then discuss common pitfalls and tips for applying the PS methods to empirical research and compare the conventional multivariable outcome regression and the two alternative PS-based methods (ie, matching and inverse probability weighting) and discuss their similarities and differences. Although we note subtle differences in causal identification assumptions, we highlight that the methods are distinct primarily in terms of the statistical modeling assumptions involved and the target population for which exposure effects are being estimated.

## INTRODUCTION

One of the central goals of clinical and epidemiologic research is to estimate the effects of exposure (eg, medical treatment, clinical practice, lifestyles and health behaviors, socioeconomic factors, and environmental factors) on population health.^1^ While randomized control trials’ role remains critical in assessing causal effects, causal inference based on observational data is essential and has evolved as an alternative approach to generating scientific evidence.^2^

Propensity score (PS) methods are among the most popular approaches for causal inference in clinical and epidemiologic research.^3^ The PS methods, as any statistical method does, have advantages and disadvantages and should be used when the application is helpful. However, confusions and misunderstanding appear to remain concerning what the PS methods can or cannot do compared to the conventional multivariable regression approach and when they become advantageous. Moreover, the differences and relative advantages of each alternative PS method are often not sufficiently understood among applied users, although such understanding can facilitate the choice of the most appropriate PS-method.

Thus, this paper aims to offer an accessible overview of causal inference using the PS methods and address some common pitfalls and provide tips for applied users. Specifically, this paper is structured as follows. First, we will give a brief overview of causal inference based on observational data. We divide causal inference into three steps and discuss what decisions and assumptions need to be made in each step. Second, we introduce how PS works in causal inference and provide a concise review of the two common methods that use PS—matching and inverse probability weighting (IPW). Third, we discuss the “pitfalls and tips” of the PS methods focusing on 1) the application of the PS methods, 2) comparison of the PS methods in general and a conventional multivariable regression model, and 3) comparison of PS matching and IPW.

## BACKGROUND ON THE TOPIC

### Basics of causal inference

Under the influential potential outcome framework proposed by Rubin,^4^ the causal effect of an exposure on an outcome is defined as follows:

$$E[Y^{a}-Y^{a^{*}}]$$

where *a* and $a^{*}$ are the different levels of exposure *A*, and *Y^a^* is a potential outcome under *A* = *a* (ie, the value of outcome that would have been observed had the person received *A* = *a*, potentially contrary to the fact). In this paper, we will focus on a binary treatment. That is, we will discuss the PS methods as a tool for estimating *E*[*Y^a^*^=1^ − *Y^a^*^=0^], where *a* = 1 and *a* = 0 indicate being exposed and unexposed, respectively. The goal of causal inference is to make inferences about this unobservable causal effect using statistical associations with a series of (often unverifiable) assumptions. By simplifying the guideline proposed previously,^5^ we will divide causal inference into the following three steps; 1) specifying causal estimand, 2) causal identification, and 3) estimation. For each step, we will briefly review its role and discuss what assumptions and decisions need to be made. More detailed introduction to causal inference is available in eMaterials 1.

#### Step 1. Specifying causal estimand

The first step of causal inference involves defining a causal effect of interest that we wish to estimate (*causal estimand*). The key ingredient to consider in determining causal estimand is whether the goal of an analysis is to estimate an effect among everyone in the population that the study sample represents versus its sub-populations. An effect in the entire population is called a *marginal effect* (ie, average treatment effect). Effects among sub-populations are called *conditional effects* because the sub-populations are defined by conditioning on specific characteristics of the population (eg, gender). Conditional effects will diverge from the marginal effects in the presence of effect measure modification by characteristics defining the sub-population.^6^^,^^7^ Because different analytic methods estimate either marginal or conditional effects or both, it is crucial to decide which effect is more of substantive interest before selecting an analytic approach.

#### Step 2. Identification

Once we define a target causal estimand, we need to consider what assumptions are required to link the unobservable causal effect of interest to observable statistical associations and whether the assumptions hold with the data at hand. This process is called causal *identification*. There are three key assumptions for identification: exchangeability, consistency, and positivity.^2^

(Marginal) *exchangeability* assumption, $Y^{a}⊥\;\;\;⊥A$ (*a* = 0, 1), means that the exposed group (*A* = 1) and the unexposed group (*A* = 0) have the same distribution of potential outcome that would be observed if everyone was exposed (*Y^a^*^=1^) and, similarly, of *Y^a^*^=0^. Exchangeability implies that the exposed versus the unexposed share equal distributions of outcome predictors, but such a condition is generally violated in observational studies.^8^^–^^10^ One may feel more confident that the groups are exchangeable conditional on a vector of covariates *L* (ie, within strata of the combinations of covariate values). This assumption is called *conditional exchangeability*, $Y^{a}⊥\;\;\;⊥A|L$ (*a* = 0, 1), and the core of causal inference from observational data.

Two other identifiability assumptions—consistency and positivity—often gain less attention than exchangeability but are likewise central in causal inference. First, the *consistency* assumption posits that exposure is sufficiently well-defined and does not have multiple “versions” that have different impacts on outcomes. Accessible introduction of this assumption is available elsewhere.^11^^,^^12^ Second, the *positivity* assumption means that both exposed and unexposed individuals need to be present in all sub-populations defined by the combinations of covariate values.^13^ We will discuss later how the PS methods address such positivity violations differently.

##### Linking potential outcomes to observed data

The causal estimand specified in Step 1 becomes a function of conditional expectation and/or probabilities that can statistically be estimated from observed data under the three identifiability assumptions from Step 2. Quantities that need to be estimated vary by causal estimands (eg, marginal vs conditional effect) and analytic approaches (eg, multivariable outcome regression vs PS methods). As discussed later, causal identification via the PS methods requires estimating conditional probabilities of exposure given *L*. Once we identify the conditional expectations and/or probabilities needed to quantify the causal effects of interest, the remaining task is to *estimate* those values from the observed data.

#### Step 3. Estimation

Although conditional expectations and probabilities can be estimated simply by computing stratum-specific averages when there are only a few covariates to consider, conditioning in causal inference (eg, adjustment for observed confounders) generally involves numerous variables, some of which are continuous. Conditional expectations and probabilities with many possible strata can be estimated by specifying statistical models, which is essentially what regression models do.

Statistical models allow the estimation of high-dimensional conditional expectations by making a series of modeling assumptions (eg, linearity between a continuous covariate and outcome, and no effect measure modification by covariates represented by omitted product terms). When the modeling assumptions do not hold (model misspecification), estimated conditional expectations would be generally biased. Note that these are statistical assumptions and distinct from the identifiability assumptions we discussed in Step 2.

The methods for causal inference, including the PS methods, generally make different modeling assumptions because they use different conditional expectations and probabilities to quantify a causal effect of interest. Thus, to understand the differences between the PS methods, it is crucial to be mindful of the statistical models that each analytic approach involves and their underlying assumptions.

### Basics of propensity scores

A PS is a conditional probability of receiving a treatment/exposure given a set of covariates:

PS=Pr[A=1|L]

The key property of PS is that exchangeability will hold conditional on PS (ie, $Y^{a}⊥\;\;\;⊥A|PS$) when the conditional exchangeability $Y^{a}⊥\;\;\;⊥A|L$ (*a* = 0, 1) holds. That is, if conditioning on the vector of covariates *L* included in the PS estimation suffices to control for all confounding, so does conditioning on estimated PS. Because the PS-based methods can only address observed confounders *L*, they are not necessarily advantageous in terms of confounding adjustment compared to conventional regression analysis.

PS is estimated by specifying a propensity model (ie, a model for an exposure), typically via logistic regression. For example, the following logistic regression can provide estimates of PS ($\hat{PS}$).

$$logitPr[A=1|L]=\alpha_{0}+L\alpha^{\prime }$$

$$\hat{PS}=\text{expit}(\hat{\alpha_{0}}+L{\hat{\alpha }}^{\prime })$$

Other specifications (eg, adding product terms) are available for the propensity model. PS can even be estimated non-parametrically (eg, via machine learning algorithms).^14^ PS can also be estimated for non-binary treatment, although a propensity model for a non-binary exposure is generally more complex and makes stronger assumptions.^15^ In estimating PS, missing data need to be handled as it is in a multivariable outcome regression. Imputing missing data is generally recommended when the missing at random assumption is plausible because ignoring missing data can result in selection bias.^16^^,^^17^

After estimating PS, there are several alternative approaches to control for the estimated PS. These approaches include stratification, regression adjustment, matching, and inverse probability weighting (IPW). We provide R and SAS codes to implement the alternative PS methods in eMaterials 2. This article will focus on matching and IPW because they are by far the most commonly used approaches; however, we provided discussions on PS stratification and regression adjustment in eMaterials 3.

### Matching

PS matching (PSM) creates pairs of the exposed and the unexposed subjects with similar estimated PS.^18^ Several approaches exist to select such pairs and have been described with details elsewhere.^19^ PSM excludes observations from individuals with extremely large or small PS if they lack corresponding pairs. In the resulting sample of the matched pairs, the exposed and unexposed groups are expected to have comparable distributions of PS and observed confounders that were used in PS estimation. In the matched sample, one can make a simple comparison of the outcome among the exposed versus the unexposed individuals:

$$E_{\text{matched}}[Y|A=1]-E_{\text{matched}}[Y|A=0],$$

where *E_matched_*[*Y*|*A* = *a*] is the conditional outcome expectation given *A* = *a* among the population that the matched sample represents. With the identifiability assumptions, this difference in the conditional expectations corresponds to the causal effect:

$$E_{\text{matched}}[Y^{a=1}-Y^{a=0}]$$

Notably, the target population of interest changes after excluding individuals in the extreme tails of the PS distribution.^20^ Specifically, PSM estimates the exposure effect in the population that the matched sample represents (ie, a population where nobody would always or never be exposed). If the distributions of effect modifiers in the matched sample differed from those in the original sample, the two marginal effects would diverge.

### Inverse probability weighting (IPW)

IPW for an exposure variable (ie, inverse probability of treatment weighting [IPTW]) is an alternative use of PS to adjust for confounding.^21^^,^^22^ Weights for IPW are typically defined as a function of PS:

• $\frac{1}{Pr[A=1|L]}=\frac{1}{PS}$ for the exposed individuals with *A* = 1
• $\frac{1}{Pr[A=0|L]}=\frac{1}{1-PS}$ for the unexposed individuals with *A* = 0.

IPW essentially duplicates observations from individuals with large weights to create a pseudo-population in which probabilities of receiving the exposure *A* do not depend on the covariates *L* included in the PS estimation. For example, assume that male gender is associated with higher probabilities of both smoking (exposure) and mortality (outcome). That is, gender confounds the association between smoking and mortality. IPW addresses such confounding by assigning larger weights to female smokers and male non-smokers so that the resulting pseudo-population has a comparable gender ratio among smokers and non-smokers. Because there is no confounding by the measured covariates *L* in the pseudo population, one can simply compare the weighted averages of the outcome for the exposed and the unexposed individuals.^22^

Alternatively, IPW can be done using an outcome regression.^21^ Weighted regression via IPW is equivalent to fitting a regression model to the pseudo-population created by the IPW. Consider the following weighted linear regression model.

$$E_{\text{pseudo}}[Y|A]=\theta_{0}{}^{*}+\theta_{1}{}^{*}A$$

where *E_pseudo_*[*Y*|*A*] is the weighted average of the outcome *Y* given *A* (ie, conditional outcome expectation in the pseudo-population). With the identifiability assumptions, this regression model approximates the model for counterfactual outcomes (ie, marginal structural model).

$$E[Y^{a}]=\theta_{0}+\theta_{1}a$$

In this marginal structural model, *θ*_1_ corresponds to the marginal effect *E*[*Y^a^*^=1^ − *Y^a^*^=0^]. IPW can also estimate conditional exposure effects, *E*[*Y^a^*^=1^ − *Y^a^*^=0^|*V*], by incorporating the information of the effect modifier *V* in weight calculation and a weighted regression model.^21^ IPW generally makes fewer assumptions for outcome models than multivariable regression does because it does not condition on numerous confounders. In fact, the weighted outcome model in IPW for marginal effect is saturated (ie, no model misspecification) when exposure is binary and time-fixed, which is the case in most IPW applications.

## PITFALLS AND TIPS

We will first discuss pitfalls and tips on the application of the PS-based methods for applied users. The subsequent section compares the PS methods with a conventional multivariable regression approach and discusses their relative advantages. The last section summarizes the differences and similarities between PS matching and IPW. Comparison with other PS-based methods (ie, stratification and regression adjustment) is available in eMaterials 4. Table 1 summarizes the discussion points. See eTable 1 for the comparison with PS stratification and regression adjustment.

**Table 1.** Comparison of multivariable regression, propensity score matching, and inverse probability weighting by the underlying assumptions

Analytic Approach	Features			

	Causal Estimand	Identifiability Assumptions	Residual Confounding/Positivity Violations	Model Specifications
Multivariable Regression	• Conditional effects within the covariate strata • Marginal effect assuming no effect measure modification by any of the measured covariates or via standardization	• Conditional exchangeability based on the covariates used in the outcome model • Consistency for the exposure of interest • Positivity conditional on the covariates	• Positivity violation if only exposed or unexposed individuals are present within the covariate strata	• Outcome model conditional on an exposure and measured covariates
PS Matching	• Marginal effect in the population represented by the matched sample, which excludes individuals with extreme PS values from the original sample.	• Conditional exchangeability based on the covariates used in the PS estimation • Consistency for the exposure of interest • Positivity conditional on the PS.	• Potential residual confounding due to wide caliper distance • Positivity is ensured by excluding unmatched individuals.	• Propensity model conditional on measured covariates. • Outcome model can be used after matching with caution.
IPW	• Conditional effects by including an additional covariate in the weighted outcome model • Marginal effect in the target population	• Conditional exchangeability based on the covariates used in the PS estimation • Consistency for the exposure of interest • Positivity conditional on the PS	• Potential positivity violation is detected as extremely large or small weights, which can be discarded before weighting.	• Propensity model conditional on measured covariates. • IP-weighted outcome model conditional on exposure (and an additional covariate if estimating conditional effects).

IPW, inverse probability weighting; PS, propensity score.

### On the application of the PS methods

#### 1. The goal of propensity models is not to predict an exposure perfectly

The goal of the PS methods is to achieve balance in observed confounders; hence, an optimal propensity model is not the one that best predicts an exposure. To achieve this goal of the PS methods as a tool for confounding adjustment, applied users need to consider the following two aspects of a propensity model specification: 1) variable selection and 2) model evaluation.

**Figure 1.**
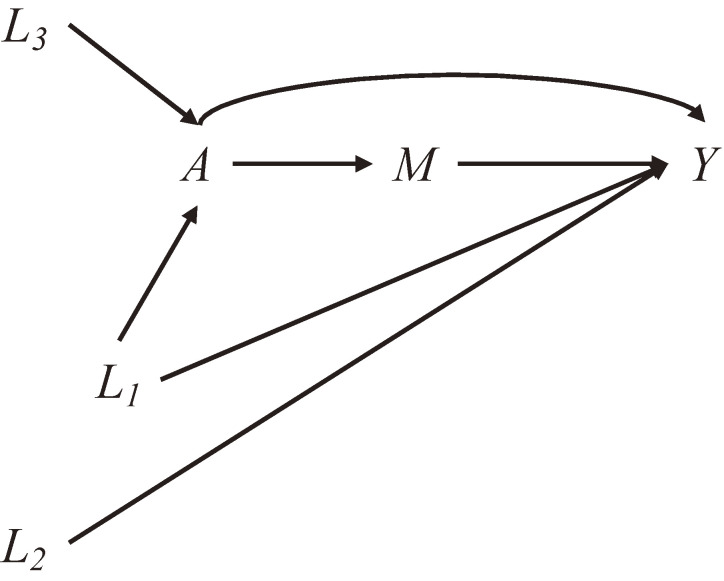
Causal Diagram Illustrating Variable Selection for Propensity Score Estimation. *A* is an exposure, *Y* is an outcome, *L*_1_, *L*_2_, and *L*_3_ are covariates, *M* is a mediator on the pathway from *A* to *Y*.

It is recommended that a propensity model include only variables that affect an outcome.^23^ If the variables also affect an exposure, they are confounders and should be adjusted for (*L*_1_ in Figure 1). Even when they do not affect exposure and, thus, are not confounders (*L*_2_ in Figure 1), their distributions are likely not identical between the exposure groups in a finite sample; adjusting for these imbalanced variables reduces bias in an effect estimate and its variance. Importantly, the “variables that affect an outcome” should be selected based on subject-matter knowledge about underlying causal structures rather than statistical associations with the outcome.^24^ Variables that affect only an exposure (*L*_3_ in Figure 1) should not be included in a propensity model because such variables can inflate the variance of the effect estimates.^25^ Moreover, variables in a propensity model should ideally be measured prior to the exposure to avoid accidentally adjusting for potential mediators (*M* in Figure 1).

Studies using the PS methods often report measures of “model fit” such as c-statistic (ie, area under the curve), aiming to evaluate the predictive performance of a propensity model. Because the prediction of exposure is not the goal of a propensity model, reporting the measures of model fit is of limited value.^26^ For instance, adding exposure predictors that are not confounders (eg, *L*_3_ in Figure 1) increases the c-statistic but does not necessarily enhance causal inference. To evaluate a propensity model in terms of confounding adjustment, covariate balance should instead be checked after PS estimation. Covariate balance can be assessed by calculating a standardized difference for each covariate using the matched sample for PSM and the weighted sample for IPW. The formulas to calculate standardized differences are available elsewhere.^3^^,^^27^ Some scholars have used <0.1 standardized difference as support for covariate balance.^28^

#### 2. PSM can suffer from residual confounding even when conditional exchangeability holds

It is generally hard to find pairs with identical PS values because PS is a continuous variable by definition and can take any value between 0 and 1. Thus, a common practice is to select an unexposed individual with a PS value closest to that of an exposed individual (nearest neighbor matching), often from a pool of unexposed individuals within a pre-specified range of PS differences from their exposed counterpart (caliper distance). Wider caliper distance may result in pairs with large differences in PS values, leading to unbalanced confounders and resulting bias in the matched sample. Austin recommends using calipers of 0.2 standard deviations of PS in the logit scale as a rule of thumb^29^; however, the optimal caliper width should ideally be determined based on the covariate balance in the matched sample.

#### 3. PSM discards unmatched observations and addresses potential positivity violations in exchange for a loss of statistical efficiency

PSM discards unmatched observations with extreme PS values. This property of PSM has an advantage and a disadvantage. The advantage is that it can explicitly address potential positivity violations.^13^ For example, individuals with extremely high PS values tend to have covariate patterns in which only exposed individuals exist. Because PSM discards information from these individuals and analyzes people within the overlapped range of the PS distribution (ie, common support), it does not rely on model extrapolation that other analytic approaches (eg, PS regression adjustment) might do. The disadvantage is that discarding information may result in imprecise estimates and loss of statistical power. Notably, discarding unmatched observations will change the target population of interest.

#### 4. Post-matching adjustment can sometimes induce bias

Although PSM can achieve balance in observed covariates in expectation, applying PSM to a finite sample sometimes results in imbalanced covariates even after matching, which can cause residual confounding. To address the residual confounding, PSM is sometimes accompanied by stratification or regression adjustment after matching.^30^ However, bias can arise if such post-matching adjustment includes variables that are not used in a propensity model.^31^

#### 5. In IPW, using stabilized weights can sometimes gain statistical efficiency

The inverse probability weights we described above had the numerator of one and are called *unstabilized* weights. Alternatively, the *stabilized* weights can be defined using other constant numbers for their numerator, often a marginal prevalence of exposure. Stabilized weights can gain statistical efficiency when a weighted outcome model makes modeling assumptions and is unsaturated (eg, a weighted outcome model for non-binary exposures or conditional effects with baseline covariates).^21^^,^^32^ When IPW was used for a binary treatment to estimate a marginal effect, a weighted outcome model would be saturated; hence, unstabilized weights and stabilized weights would give identical results. There are some cases where unstabilized weights, not stabilized weights, should be used (eg, estimating an effect of a dynamic treatment regimen), but these cases are beyond the scope of this introductory paper.^33^

#### 6. IPW can be used to address selection bias too

**Figure 2.**
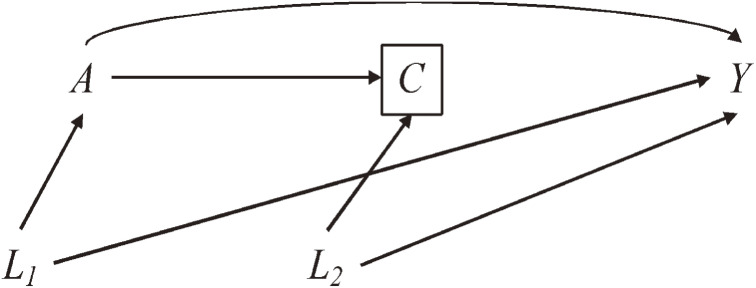
Causal Diagram Illustrating Confounding and Selection Bias. *A* is an exposure, *Y* is an outcome, *C* is a censoring status, *L*_1_ is a vector of common causes of *A* and *Y*, and *L*_2_ is a vector of common causes of *C* and *Y*. *L*_1_ confounds the association between *A* and *Y*. When the uncensored sample (*C* = 0) is analyzed, the analysis is effectively conditioning on *C* = 0 (ie, a collider) and inducing selection bias through *L*_2_.

IPW can also address selection bias.^34^ Inverse probability weights for censoring (IPCW) are calculated based on probabilities of selection/censoring conditional on exposure and common causes of the censoring and the outcome. Note that the censoring weights can incorporate variables that are not confounders but cause selection bias (eg, *L*_2_ in the causal diagram in Figure 2). While IPCW is a useful tool to advance causal inference, the interpretation of the estimated effects after IPCW may not be straightforward in the presence of competing risk.^35^^–^^37^ Moreover, the weight calculation requires the information on exposure and covariates among the censored individuals, which often is unavailable.^38^

#### 7. PSM and IPW both require methods for the analysis of correlated observations

In PSM, the post-matching analysis needs to take account of the within-matched pair correlations.^39^ For example, post-matching analysis can use paired *t*-test or Wilcoxon’s rank sum test for continuous outcomes, and McNemar’s test and conditional logistic regression for binary outcomes, and cox proportional hazards regression for survival outcomes.^39^^,^^40^ Similarly, in IPW, standard errors from the IP-weighted outcome regression need to be corrected due to the dependent observations in the weighted data; using robust variance or non-parametric bootstrapping is recommended to estimate standard errors (R and SAS codes are available in eMaterials 2).^32^

### PS methods versus multivariable outcome regression

#### 8. The PS methods and multivariable outcome regression both assume no unmeasured confounding. However, there are properties of the PS methods that are sometimes advantageous

The PS methods and the multivariable outcome regression approach both assume conditional exchangeability given measured covariates. Thus, they can only address confounding caused by measured covariates and are equally prone to bias due to unmeasured confounders. Nevertheless, the PS methods can sometimes be preferable for the following five reasons.

First, in theory, the PS methods can result in data analysis with more integrity and work against p-hacking.^41^ Most of the PS methods’ modeling decisions come *before* looking at outcome data. Thus, investigators may be less tempted to change model specifications to make the results align with their expectations. In PSM, for instance, the investigator first specifies a propensity model and estimate PS, creates a matched sample, checks the balance of observed covariates between the exposed and the unexposed, and, if unbalanced, goes back and re-specifies a propensity model, all of which can be done without outcome data. Even for the methods that specify an outcome model (ie, regression adjustment and IPW), the outcome model generally makes fewer or even no modeling assumptions than a multivariable outcome regression conditioning on numerous covariates. However, this first advantage may not fully be leveraged in the applied research because careless application of the PS methods would not yield this theoretical property.

Second, potential positivity violations tend to become more visible in the PS methods because extreme PS values can signal covariate patterns in which only the exposed or the unexposed are present. As we describe below, the PS methods handle potential positivity violations differently.

Third, when the outcome is rare, conditioning on numerous covariates via a multivariable regression can result in imprecise estimation. When the exposure is non-rare, the PS methods can work better for rare outcomes because they convert the high-dimensional covariates into a single variable, PS.

Fourth, the PS methods and the multivariable outcome regression make qualitatively different modeling assumptions. The PS methods’ primary modeling decisions are for a propensity model. Although the propensity models and outcome models conditional on measured covariates are both prone to misspecification, one may feel more confident of correctly specifying an exposure model in situations where more knowledge about the relationships with covariates is available for exposure than for an outcome. A doubly-robust method (eg, targeted maximum likelihood estimation) can accommodate both models and consistently estimate conditional expectancies of interest if either a propensity model or an outcome model is correctly specified.^42^^,^^43^ Notably, multivariable outcome regression technically estimates *conditional* effects within the strata of observed covariates. Although a marginal effect can rigorously be estimated via standardization (R and SAS codes are available in eMaterials 2),^44^ a more common approach for estimating marginal effects with multivariable outcome regression is to assume no effect measure modification by ANY of measured covariates (ie, no product term between exposure and covariates). On the other hand, the PS methods tend to make no or fewer assumptions for effect measure modification, although they instead make assumptions for a propensity model.

Lastly, IPW can be expanded to causal inference for a time-varying exposure in the presence of time-varying confounding.^21^^,^^45^ Conventional analytic approaches, including other PS methods, fail to estimate the effects of a time-varying exposure when prior exposure affects confounders of subsequent exposure.^46^

### Comparison of PSM and IPW

#### 9. The alternative PS methods rely on the same assumptions for exchangeability and consistency but deal with the positivity assumption differently

Both PSM and IPW rely on the same identifiability assumptions of conditional exchangeability and consistency. In contrast, these methods take different approaches to handle potential positivity violations. In IPW, individuals will receive substantially large or small weights when their covariate patterns potentially violate positivity. Trimming such observations with extreme weights is often recommended.^47^ On the other hand, PSM explicitly addresses potential positivity violations by excluding those who have extreme PS values and, thus, cannot be matched (so-called “off-support” individuals). While such explicit handling of positivity violations is the advantage of the PS methods, one caveat is that causal estimand of interest generally changes after excluding individuals who potentially violate positivity.^20^

#### 10. Although the PS methods both make the same exchangeability assumption, PSM can suffer from residual confounding

Both PSM and IPW are based on the same conditional exchangeability (ie, no confounding conditional on measured covariates). However, as we noted in #2 above, PSM may result in an insufficient balance of the measured covariates when the pre-specified caliper is wide. On the other hand, IPW does not suffer from residual confounding, assuming the models involved are correctly specified.

#### 11. The PS methods make different modeling assumptions after propensity score estimation

Both PSM and IPW specify a propensity model to estimate PS. PSM often does not require any further modeling once PS is estimated although an outcome model is sometimes used to make post-matching adjustment. IPW specifies a weighted outcome model to approximate a marginal structural model, but the outcome model tends to make fewer assumptions than it does in PSM (when post-matching adjustment via regression is conducted) or even be saturated (no modeling assumption) when estimating the marginal effect of a single-point binary exposure.

#### 12. The PS methods target different causal estimands (ie, each method answers a different research question)

PSM and IPW generally target different causal estimands.^20^^,^^48^^,^^49^ In other words, when an effect estimate from one PS method differs from an estimate from another PS method, they can both be correct but simply answer different questions. PSM estimates a marginal effect in a population represented by a matched sample. Because the matched sample excludes individuals with extreme PS values, PSM does not estimate an exposure effect among individuals who would always or never be exposed unless they were intervened and forced to have an alternative exposure level. PSM often uses all exposed individuals and matches them with their unexposed pairs. This approach will estimate an exposure effect among the people who were in fact exposed (ie, average treatment effect in the treated).^3^ IPW can estimate both marginal and conditional effects, depending on the definition of weights and specification of a marginal structural model.

### Summary

Causal inference using PS-based methods has become common, but the increasing popularity of the PS methods might have been driven, at least partly, by the hype that the seemingly “sophisticated” approach somehow leads to more rigorous analysis. The PS methods should be used when their applications are truly beneficial (eg, high-dimensional covariates for a rare outcome). Moreover, even when using the PS methods is justified, we argue that choosing an optimal PS-based approach requires a good understanding of the underlying decisions and assumptions that each PS method makes.

The underlying identifiability assumptions are largely comparable across the methods (ie, multivariable outcome regression and the alternative PS-methods) although we noted some subtle differences concerning residual confounding and positivity violation. We argue that the alternative methods differ primarily in terms of modeling assumptions that they make and, most importantly, causal estimand (ie, they estimate effects in different target populations and, thus, answer different research questions). We recommend the applied users decide which analytic approach to use considering the relative advantages and disadvantages discussed in this article.

## APPENDIX A. SUPPLEMENTARY DATA

eMaterials 1. More comprehensive overview of basics of causal inference

eMaterials 2. R and SAS code for multivariable regression, standardization, and alternative PS methods

eMaterials 3. Overview of propensity score stratification and regression adjustment

eMaterials 4. Comparison of propensity score stratification, regression adjustment, matching, and inverse probability weighting

eTable 1. Comparison of alternative analytic methods by the underlying assumptions
