# Supplementary material for: Using Propensity Scores for Causal Inference: Pitfalls and Tips
Source: J Epidemiol. 2021 Aug 5;31(8):457–63. doi: 10.2188/jea.JE20210145 (PMC8275441; doi:10.2188/jea.JE20210145)
Supplement: Supplementary file 1 [file je-31-457-s001.pdf]

eMaterials 1. More comprehensive overview of basics of causal inference

Under the influential potential outcome framework proposed by Rubin[1], the causal effect of an exposure on an outcome is defined as follows:

$$E[Y^a - Y^{a^*}]$$

where a and a^* are the different levels of an exposure A, and $Y^a$is a potential outcome under $A = a$ (i.e., the value of outcome that would have been observed had the person received A=a, potentially contrary to the fact). In this paper, we will focus on a binary treatment. That is, we will discuss the PS methods as a tool for estimating $E[Y^{a=1} - Y^{a=0}]$, where $a = 1$ and $a = 0$ indicate being exposed and unexposed, respectively. Causal effects can be defined for categorical and failure-time outcomes and in the relative scale (e.g., risk ratio for a binary outcome).

The fundamental challenge in causal inference is that observed data contains only one of the two potential outcomes (i.e., $Y^{a=1}$for the exposed individuals with $A = 1$ and $Y^{a=0}$for the unexposed individuals with $A = 0$). The other potential outcome is *missing* and never observed simultaneously for the same individual (i.e., $Y^{a=1}$for the unexposed individuals with $A = 0$ and $Y^{a=0}$for the exposed individuals with $A = 1$). The goal of causal inference is to make inferences about the unobservable causal effect using statistical associations with a series of (often unverifiable) assumptions. By simplifying the guideline proposed previously[2], we will divide causal inference into the following three steps; 1) specifying causal estimand, 2) causal identification, and 3) estimation. For each step, we will briefly review its role and discuss what assumptions and decisions need to be made.

Step 1. Specifying causal estimand

The first step of causal inference involves defining a causal effect of interest that we wish to estimate (*causal estimand)*. In other words, we need to specify what *questions* we aim to answer using the causal inference framework. Determining causal estimand requires specification of a target population for inference (i.e., a group of people for which we want to estimate the exposure effect). The key ingredient to consider in determining a target population is whether the goal of an analysis is to estimate an effect among everyone in the population that the study sample represents vs. its sub-populations. Imagine we collected data from a nationally representative sample. We could use this data to estimate an exposure effect of interest among *everyone* in the sample and the population it represents (i.e., $E[Y^{a=1} - Y^{a=0}]$; the difference between expected outcomes had everyone in the country been exposed vs. unexposed). This effect in the entire population is called a *marginal effect* (a.k.a., average treatment effect; ATE). We could also estimate the effect among men and women, separately (i.e., $E[Y^{a=1} - Y^{a=0}|gender = men]$ and $E[Y^{a=1} - Y^{a=0}|gender = women]$). These gender-specific effects are defined for the sub-populations conditional on one's gender and, hence, called *conditional effects*. Marginal effects and conditional effects may or may not differ in size. When the conditional effects differ by gender (i.e., effect modification by gender), marginal effects will diverge from the conditional effects.[3,4] Because different analytic methods estimate either marginal or conditional effects or both, it is crucial to decide which effect is more of substantive interest before selecting an analytic approach.

Step 2. Identification

Once we define a target causal estimand, we need to consider what assumptions are required to link the unobservable causal effect of interest to observable statistical associations

and whether the assumptions hold with the data at hand. This process is called causal *identification*. There are three key assumptions for identification: exchangeability, consistency, and positivity.[5]

Exchangeability

(Marginal) *exchangeability* assumption, $Y^a \perp\!\!\!\perp A$ (a = 0, 1), implies that the treated vs. the untreated share equal distributions of outcome predictors and would have the same outcomes if their exposure levels were the same. In observational studies, however, the exposed group generally have different backgrounds than the unexposed group do (a.k.a. confounding and selection bias, depending on the causal structure that led to the differential distributions of the background factors) and, hence, marginal exchangeability is unlikely to hold.[6–8]

One may feel more confident that the groups are exchangeable conditional on some covariates (i.e., within strata of the combinations of covariate values). For example, the exposed and the unexposed may not be exchangeable in the entire study sample because of confounding by age. Yet, the two groups may become exchangeable if they were compared among people of the same age. Such condition is called *conditional exchangeability*, $Y^a \perp\!\!\!\perp A|L$ (a = 0, 1), and the core of causal inference from observational data. This assumption is also called “no unmeasured confounding assumption” or “ignorability” in literature.[7] Any causal inference methods based on confounding adjustment rely on the assumption that the observed L suffices to achieve conditional exchangeability. Approaches to select such a set of L have been discussed elsewhere.[9,10] These approaches’ critical implication is that the selection of covariates requires subject-matter knowledge of the underlying causal structure for the exposure-outcome relationship in question and cannot be done solely by statistical and data-driven approaches.[11]

Consistency and Positivity

Two other identifiability assumptions — consistency and positivity — often gain less attention than exchangeability but are central in causal inference too. First, the *consistency* assumption, $Y^a = Y^A = Y \; when \; A = a$, pertains to an exposure's definition and operationalization. This assumption means that one's potential outcome under the level of exposure they received ($Y^a \; when \; A = a$) is equal to their observed outcome ($Y^A = Y$). This seemingly obvious assumption does not hold when exposure is ill-defined and has multiple "versions" that might have different impacts on outcomes. With multiple versions of an exposure A, causal inference is not intuitive because the corresponding potential outcome Y^a will be ill-defined too. Accessible introduction of this assumption is available elsewhere.[12,13]

Second, the *positivity* assumption pertains to the distribution of exposure in the target population and the data at hand. Positivity requires that, for all possible combinations of covariates in the population, the probability of being exposed must be strictly between 0 and 1. More informally, positivity means that both exposed and unexposed individuals need to be present in all sub-populations defined by the combinations of covariate values.[14] In practice, positivity violation can occur empirically when there are too many covariates relative to the sample size or when the covariate set contains a continuous variable. That is, for a finite sample, some covariate strata may end up consisting of all exposed or unexposed individuals by chance as the number of the covariate value combinations increases. We will discuss later how the PS methods address such positivity violations differently.

Linking potential outcomes to observed data

The causal estimand specified in Step 1 becomes a function of conditional expectation and/or probabilities that can statistically be estimated from observed data under the three

identifiability assumptions from Step 2. Depending on the causal estimand and analytic approach, different quantities will need to be estimated. For example, a conditional effect among individuals with $L = l$, $E[Y^{a=1} - Y^{a=0} | L = l]$, will be identified as the difference in two conditional expectations of the outcome ($E[Y|A = 1, L = l] - E[Y|A = 0, L = l]$), assuming conditional exchangeability given L, consistency, and positivity. As we will discuss later, causal identification via the PS methods instead requires estimating conditional probabilities of exposure given L. Once we identify the conditional expectations needed to quantify the causal effects of interest, the remaining task is to *estimate* these values from the observed data.

Step 3. Estimation

With only a few covariate patterns to consider, conditional expectations and probabilities can be estimated simply by computing stratum-specific averages. However, conditioning in causal inference (e.g., adjustment for observed confounders) generally involves numerous variables, some of which are continuous. Thus, the number of possible combinations of covariate values is substantially large, making it often impossible to manually estimate stratum-specific averages with a finite sample.

Conditional expectations and probabilities with many possible strata can be estimated by specifying statistical models, which is essentially what regression models do. For example, consider the following regression model.

$$E[Y|A, L] = \beta_0 + \beta_1 A \; + L\beta'$$

In this model, β_1 corresponds to $E[Y|A = 1, L = l] - E[Y|A = 0, L = l]$, the conditional effect of the exposure A within the strata defined by L under the identifiability assumptions.

Statistical models allow the estimation of high-dimensional conditional expectations by making a series of modeling assumptions (e.g., linearity between a continuous covariate and

outcome, and no effect modification by covariates represented by omitted product terms). When the modeling assumptions do not hold (model misspecification), estimated conditional expectations would be generally biased. Note that these are statistical assumptions and distinct from the identifiability assumptions we discussed in Step 2. Unbiased estimates of conditional expectations from correctly specified models will not have causal interpretation unless the identifiability assumptions hold.

The methods for causal inference, including the PS methods, generally make different modeling assumptions because they use different conditional expectations and probabilities to quantify a causal effect of interest. Thus, to understand the differences between the PS methods, it is crucial to be mindful of the statistical models that each analytic approach involves and their underlying assumptions.

eMaterials 2. R and SAS code for multivariable regression, standardization, and alternative PS methods

We provide a sample code to implement the PS methods in R and SAS using the simulated data.

In this example, we used the following R packages:

```
library(tidyverse)
library(broom)
library(gtsummary)
library(MatchIt)
library(geepack)
library(boot)
```

The simulated data was generated by the R code shown below.

```
set.seed(0)
n.obs = 10000   #set sample size

#---- True parameters in outcome model ----
b0 = 60
b1 = 5
b2 = -0.3
b3 = -0.1
b4 = 8
b5 = 3
b6 = 2

#---- True parameters in exposure odds model ----
g0 = log(0.20/(1-0.20))
g1 = log(1.01)
g2 = log(1.005)
g3 = log(0.6)
g4 = log(0.5)
g5 = log(0.8)
```

```r
#Function to compute outcome values
## Use the parameters specified above
mean_out <- function(C1, C2, C3, exposure){
  b0 + b1*exposure + b2*C1 + b3*I(C1^2) + b4*C2 + b5*C3 + b6*exposure*C2 + rn
orm(n = n.obs, mean = 0, sd = 5)
}


#Function to compute exposure probabilities
## Use the parameters specified above
prob_exp <- function(C1, C2, C3){
  exp(g0 + g1*C1 + g2*I(C1^2) + g3*C2 + g4*C3 + g5*C2*C3)/(1 + exp(g0 + g1*C1
 + g2*I(C1^2) + g3*C2 + g4*C3 + g5*C2*C3))
}


#Simulate the data
df.sim <- tibble("ID" = seq(from = 1, to = n.obs, by = 1),
                 "C1" = rnorm(n  = n.obs, mean = 0, sd = 5),
                 "C2" = rbinom(n = n.obs, size = 1, p = 0.4),
                 "C3" = rbinom(n  = n.obs, size = 1, p = 0.3),
                 "Pexposure" = prob_exp(C1, C2, C3),
                 "Exposure" = rbinom(n = n.obs, size = 1,
                                     prob = Pexposure),
                 "Outcome" = as.numeric(mean_out(C1,C2,C3, Exposure)))
```

In this simulated data, A is exposure, C_1 is a continuous covariate, and C_2 and C_3 are binary covariates.

The true outcome model is specified as follows:

$$Y = \beta_0 + \beta_1 A + \beta_2 C_1 + \beta_3 C_1^2 + \beta_4 C_2 + \beta_5 C_3 + \beta_6 A * C_2 + \epsilon$$

$$\epsilon \sim \ N(0, 5^2)$$

The true propensity model is specified as follows:

$$logit(Pr[A = 1 \,|\, C_1, C_2, C_3]) = \gamma_0 + \gamma_1 C_1 + \gamma_2 C_1^2 + \gamma_3 C_2 + \gamma_4 C_3 + \gamma_5 C_2 * C_3$$

True causal effect of exposure A among those with $C_2 = 0$ is $\beta_1 = 5$. True causal effect of exposure A among those with $C_2 = 1$ is $\beta_1 + \beta_6 = 5 + 2 = 7$. The marginal effect is $5*0.6 + 7*0.4 = 5.8$ because 40% of the total population has $C_2 = 1$.

Multivariable regression

```
        # Correctly specified model
df.sim %>%
  lm(Outcome ~ Exposure*C2 + C1 + I(C1^2) + C3, data = .) %>%
  tidy(conf.int = TRUE)
```

The estimate for Exposure is 5.02. Note that this is an estimate of the conditional effect for $C_2 = 0$ and it is identical to the true value $\beta_1 = 5$ because the model is correctly specified. The conditional effect for $C_2 = 1$ is estimated to be 5.02 + 1.93 = 6.95, which again is nearly identical to the true parameter $\beta_1 + \beta_6 = 5 + 2 = 7$.

```
## # A tibble: 7 x 7
##   term        estimate std.error statistic   p.value conf.low conf.high
##   <chr>          <dbl>     <dbl>     <dbl>     <dbl>    <dbl>     <dbl>
## 1 (Intercept)   60.0      0.0875      686.        0.        59.8      60.2
## 2 Exposure       5.02     0.166        30.2   4.61e-192    4.69      5.34
## 3 C2             8.10     0.112        72.4       0.        7.88      8.32
## 4 C1            -0.305    0.0101      -30.1   2.49e-190   -0.324    -0.285
## 5 I(C1^2)       -0.100    0.00148     -67.8       0.       -0.103   -0.0972
## 6 C3             2.94     0.111        26.5   4.31e-150    2.72      3.16
## 7 Exposure:C2    1.93     0.294         6.56  5.77e- 11    1.35      2.51
```

The misspecified model assuming the linearity for C_1 and no interaction between the exposure and C_2 yields the biased estimate for exposure effect of 4.88.

```
# Misspecified model
df.sim %>%
```

```r
  lm(Outcome ~ Exposure + C1 + C2 + C3,
                     data = .) %>%
  tidy(conf.int = TRUE, exp = TRUE)
```

```
## # A tibble: 5 x 7
##   term        estimate std.error statistic   p.value conf.low conf.high
##   <chr>          <dbl>     <dbl>     <dbl>     <dbl>    <dbl>     <dbl>
## 1 (Intercept)   57.6      0.0954     604.  0.            57.4      57.8
## 2 Exposure       4.88     0.167       29.3 6.26e-181     4.55       5.20
## 3 C1            -0.312    0.0123     -25.4 1.79e-138    -0.336    -0.288
## 4 C2             8.26     0.125       65.9 0.            8.01       8.50
## 5 C3             2.90     0.134       21.6 3.64e-101     2.63       3.16
```

Standardization (a.k.a., g-formula/g-computation)

One can standardize the conditional effect estimates from the correctly specified multivariable regression model to get an estimate of a marginal effect.

```r
# Make copies of original data
df.sim.a1 <- df.sim %>%
  mutate(Outcome  = NA,
         Exposure = 1) #Assign Exposure = 1 to everyone

df.sim.a0 <- df.sim %>%
  mutate(Outcome  = NA,
         Exposure = 0) #Assign Exposure = 0 to everyone

df.sim.combined <-
  bind_rows(df.sim.a1,df.sim.a0)

# Fit an outcome model to the original data
## Correctly specified model
```

```
gcomp.fit <- df.sim %>%
  lm(Outcome ~ Exposure*C2 + C1 + I(C1^2) + C3, data = .)

# Predict outcome values using the copied datasets
df.sim.combined$pred <- predict(gcomp.fit, newdata = df.sim.combined)

# ATE Estimate: Difference between mean predicted values for rows with A=1 and m
ean predicted values for rows with A = 0
df.sim.combined %>%
  group_by(Exposure) %>%
  summarise(
    mean.Y = mean(pred)
  ) %>%
  pivot_wider(
    names_from = Exposure,
    names_glue = "mean.Y.{Exposure}",
    values_from = mean.Y
  ) %>%
  mutate(
    ATE = mean.Y.1-mean.Y.0
  )
```

The resulting estimate of a marginal effect is 5.78 — this is a consistent estimate of the true marginal effect of 5.8.

```
## # A tibble: 1 x 3
##   mean.Y.0 mean.Y.1   ATE
##      <dbl>    <dbl> <dbl>
## 1     61.6     67.4  5.78
```

Confidence intervals for the standardized estimate can be obtained via bootstrapping.

```
standardization.boot <- function(data, indices){
  df <- data[indices,]
  df.a1 <- df %>%
```

```
  mutate(Outcome  = NA,
         Exposure = 1)

  df.a0 <- df %>%
    mutate(Outcome  = NA,
           Exposure = 0)

  df.combined <-
    bind_rows(df.a1,df.a0)

  gcomp.fit <- df %>%
    lm(Outcome ~ Exposure*C2 + C1 + I(C1^2) + C3, data = .)
  df.combined$pred <- predict(gcomp.fit, newdata = df.combined)
  output <- df.combined %>%
    group_by(Exposure) %>%
    summarise(
      mean.Y = mean(pred)
    ) %>%
    pivot_wider(
      names_from = Exposure,
      names_glue = "mean.Y.{Exposure}",
      values_from = mean.Y
    ) %>%
    mutate(
      ATE = mean.Y.1-mean.Y.0
    )
  return(output$ATE)
}

# bootstrap
standardization.results <- boot(data=df.sim, statistic=standardization.boot,
R=100) # 100 bootstrapped samples

# generating confidence intervals
```

```r
empirical.se <- sd(standardization.results$t) # get empirical standard error estimate
estimate <- standardization.results$t0
ll <- estimate - qnorm(0.975)*empirical.se # normal approximation
ul <- estimate + qnorm(0.975)*empirical.se

data.frame(cbind(estimate, empirical.se, ll, ul))
```

```
##   estimate empirical.se       ll       ul
## 1 5.782279    0.1428676 5.502264 6.062294
```

<u>Propensity Score Estimation</u>

```r
# Fit a propensity model
## Correct model
PS.fit.correct <-
  df.sim %>%
  glm(Exposure ~ C1 + I(C1^2) + C2*C3, family = "binomial", data=.)
## Misspecified model
PS.fit.misspecified <-
  df.sim %>%
  glm(Exposure ~ C1 + C2 + C3, family = "binomial", data=.)

# Estimate PS
df.sim$PS.correct <-
  predict(PS.fit.correct, type = "response")

df.sim$PS.misspecified <-
  predict(PS.fit.misspecified, type = "response")
```

PS Method 1: Propensity Score Stratification

Using deciles, we estimate ten stratum-specific effect estimates. Assuming no effect measure modification by PS strata, the ATE estimate is the average of the stratum-specific estimates (=5.65).

```
df.sim %>%
  mutate(PS.correct.strata = gtools::quantcut(PS.correct, 10)) %>% #deciles
  group_by(Exposure,PS.correct.strata) %>% #group by exposure levels and PS strata
  summarise(mean.Y = mean(Outcome)) %>% #calculate group-specific outcome means
  pivot_wider( # convert to wide format
    names_from = Exposure,
    names_glue = "mean.Y.{Exposure}",
    values_from = mean.Y
  ) %>%
  mutate(
    ATE.strata = mean.Y.1-mean.Y.0, # Calculate stratum-specific ATEs
    ATE = mean(ATE.strata) # Overall ATE = Average of stratum-specific ATEs
  )
```

```
## # A tibble: 10 x 5
##    PS.correct.strata mean.Y.0 mean.Y.1 ATE.strata   ATE
##    <fct>                <dbl>    <dbl>      <dbl> <dbl>
##  1 [0.0554,0.0729]       69.8     76.9       7.09  5.65
##  2 (0.0729,0.106]        62.7     68.5       5.87  5.65
##  3 (0.106,0.129]         63.0     67.8       4.81  5.65
##  4 (0.129,0.135]         67.2     74.6       7.45  5.65
##  5 (0.135,0.155]         65.1     71.7       6.60  5.65
##  6 (0.155,0.198]         60.5     66.0       5.44  5.65
##  7 (0.198,0.202]         59.9     64.9       5.08  5.65
##  8 (0.202,0.212]         58.8     64.1       5.31  5.65
##  9 (0.212,0.235]         57.4     62.3       4.88  5.65
```

```
## 10 (0.235,0.608]          51.9      55.9        3.99   5.65
```

PS Method 2: Propensity Score Regression Adjustment

Using PS regression adjustment, the effect estimate was 5.63. Note that this model assumes linearity for the estimated PS (PS.correct) in the outcome model and also no effect measure modification by PS.correct.

```
df.sim %>%
  lm(Outcome ~ Exposure + PS.correct, data=.) %>%
  tidy()
```

```
## # A tibble: 3 x 5
##   term        estimate std.error statistic   p.value
##   <chr>          <dbl>     <dbl>     <dbl>     <dbl>
## 1 (Intercept)    73.8      0.159     463.         0.
## 2 Exposure        5.63     0.161      34.9    6.81e-252
## 3 PS.correct    -74.2      0.914     -81.1        0.
```

PS Method 3: Propensity Score Matching

We used nearest neighbor matching with caliper width of 0.2 SD of PS in the logit scale. We also show the code to compare standardized mean differences to check covariate balance after matching.

```
fit.match <- matchit(Exposure ~ C1 + I(C1^2) + C2*C3, method = "nearest",cali
per = 0.2, distance = "linear.logit", data=df.sim)


df.matched <- match.data(fit.match) # Create a matched sample
```

```
# Check covariate balance via standardized mean differences
df.smd <- summary(fit.match,standardize = TRUE)
df.smd.p <- rbind(
  df.smd$sum.all %>% as.data.frame() %>% mutate(term = rownames(.)) %>% mutat
e(Sample = "All"),
  df.smd$sum.matched %>% as.data.frame() %>% mutate(term = rownames(.)) %>% m
utate(Sample = "Matched")
)
df.smd.p %>%
  ggplot(aes(x = `Std. Mean Diff.`, y = term, color = Sample)) +
  geom_point(size = 2) +
  geom_vline(xintercept = 0) +
  theme_bw(base_family = "serif") +
  labs(x = "Standardized Mean Difference", y = "")
```

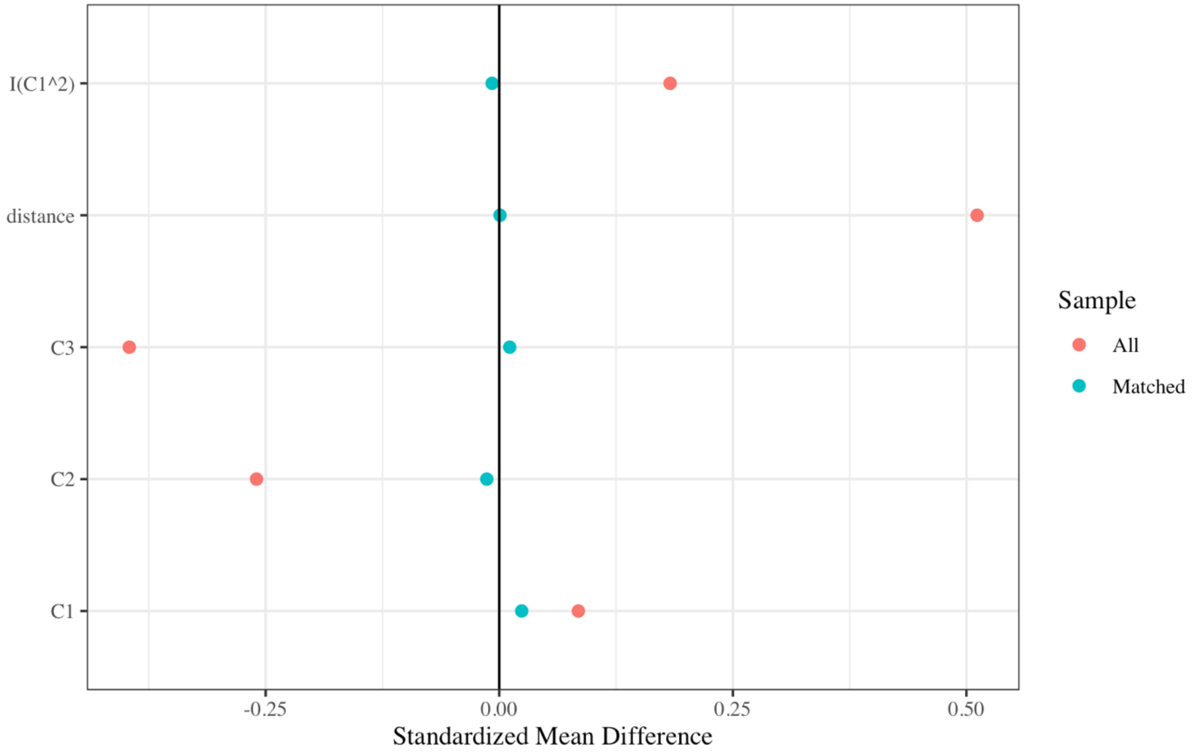


```
df.matched %>%
  lm(Outcome ~ Exposure, data=.) %>%
  tidy()
```

The estimated effect was 5.69. This is slightly different from the true ATE (=5.8), partly because the inferential target is now changed to the population that the matched sample represents, not the original population.

```
## # A tibble: 2 x 5
##   term        estimate std.error statistic  p.value
##   <chr>          <dbl>     <dbl>     <dbl>    <dbl>
## 1 (Intercept)    59.6      0.195     305.   0.
## 2 Exposure        5.69     0.276      20.6 7.56e-89
```

Using the misspecified propensity model that assumes the linearity for C_1 and no interaction between C_2 and C_3 yields the biased estimate for exposure effect of 5.33.

```
fit.match <- matchit(Exposure ~ C1 +  C2 + C3, method = "nearest",caliper =
0.2, distance = "linear.logit",data=df.sim)


df.matched <- match.data(fit.match)


df.smd <- summary(fit.match,standardize = TRUE)
df.smd.p <- rbind(
  df.smd$sum.all %>% as.data.frame() %>% mutate(term = rownames(.)) %>% mutat
e(Sample = "All"),
  df.smd$sum.matched %>% as.data.frame() %>% mutate(term = rownames(.)) %>% m
utate(Sample = "Matched")
)
df.smd.p %>%
  ggplot(aes(x = `Std. Mean Diff.`, y = term, color = Sample)) +
  geom_point(size = 2) +
  geom_vline(xintercept = 0) +
  theme_bw(base_family = "serif") +
  labs(x = "Standardized Mean Difference", y = "")
```

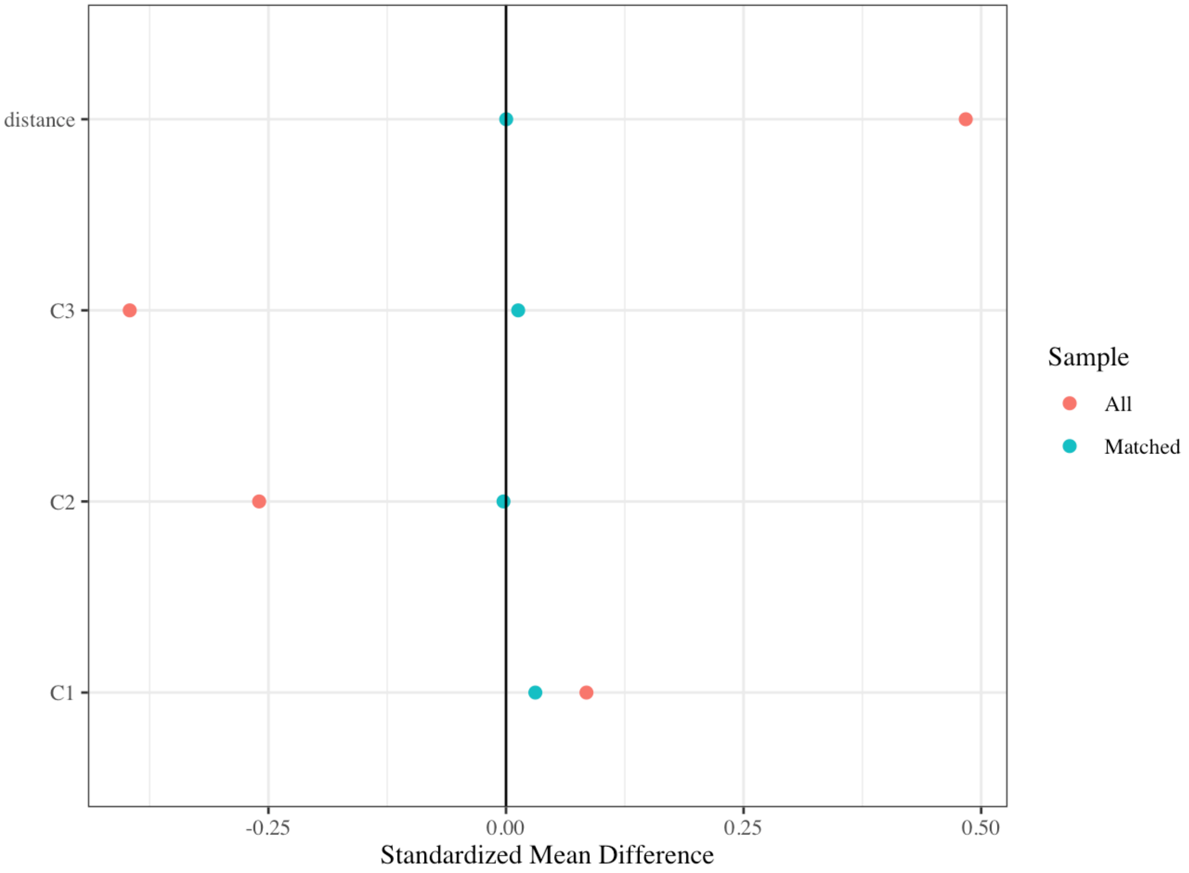


```
df.matched %>%
  lm(Outcome ~ Exposure, data=.) %>%
  tidy()
```

```
## # A tibble: 2 x 5
##   term        estimate std.error statistic  p.value
##   <chr>          <dbl>     <dbl>     <dbl>    <dbl>
## 1 (Intercept)    60.0      0.196     306.   0.
## 2 Exposure        5.33     0.277      19.2 2.56e-78
```

PS Method 4: Inverse Probability Weighting

```
df.sim <- df.sim %>%
  mutate(ustw = ifelse(Exposure == 1,
                       1/PS.correct, #Denomenator = PS when Exposure =1
                       1/(1-PS.correct))) #Denomenator = 1- PS when Exposure
= 0

# specify MSM
df.sim %>%
  geeglm(Outcome ~ Exposure, weights = ustw, data=.,id = ID) %>% #We used GEE to
 adjust for standard errors
  tidy()
```

Using the generalized estimating equation to correct the standard error estimate for correlated data, the estimated ATE was 5.83.

```
## # A tibble: 2 x 5
##   term        estimate std.error statistic p.value
##   <chr>          <dbl>     <dbl>     <dbl>   <dbl>
## 1 (Intercept)    61.6     0.0847   528318.       0
## 2 Exposure        5.83    0.240       590.       0
```

Alternatively, bootstrapping can be used to obtain a valid standard error estimate.

```
ipw.boot <- function(data, indices){
  df <- data[indices,]
  df <- df %>%
    mutate(ustw = ifelse(Exposure == 1,
                         1/PS.correct,
                         1/(1-PS.correct)))

  # fit MSM
```

```
  output <- df %>%
    lm(Outcome ~ Exposure, weights = ustw, data=.)
  return(output$coefficients[2]) # extract point estimate for Exposure
}

# bootstrap
ipw.results <- boot(data=df.sim, statistic=ipw.boot, R=100) # 100 bootstrappe
d samples

# generating confidence intervals
empirical.se <- sd(ipw.results$t) # get empirical standard error estimate
estimate <- ipw.results$t0
ll <- estimate - qnorm(0.975)*empirical.se # normal approximation
ul <- estimate + qnorm(0.975)*empirical.se

data.frame(cbind(estimate, empirical.se, ll, ul))
```

```
##          estimate empirical.se       ll       ul
## Exposure 5.832164    0.2568622 5.328724 6.335605
```

When a propensity model is misspecified, the ATE estimate from IPW was biased (= 5.12).

```
df.sim <- df.sim %>%
  mutate(ustw.misspecified = ifelse(Exposure == 1,
                          1/PS.misspecified, #Denominator = PS when Exposure =1
                          1/(1-PS.misspecified))) #Denominator = 1- PS when Expo
sure = 0

# fit MSM
df.sim %>%
  geeglm(Outcome ~ Exposure, weights = ustw.misspecified, data=.,id = ID) %>%
 #We used GEE to adjust for standard errors
  tidy()
```

```
## # A tibble: 2 x 5
##   term        estimate std.error statistic p.value
##   <chr>          <dbl>     <dbl>     <dbl>   <dbl>
## 1 (Intercept)    61.7     0.0817   570320.       0
## 2 Exposure        5.12    0.241       451.       0
```

SAS code to implement the same analyses for the same simulated dataset is shown below. Note that the results may not be perfectly identical the two languages perform slightly different calculations internally.

```
/*Multivariable regression*/
proc glm;
       model outcome = exposure c2 c1 c1*c1 c3 exposure*c2 / solution;
run; quit;
proc glm ;
       model outcome = exposure c1 c2 c3 / solution;
run; quit;


/*Standardization (a.k.a., g-formula/g-computation)*/
*Note: PROC CAUSALTRT fits separate models for exposure = 0 and 1;
proc causaltrt method=regadj;
       psmodel exposure(ref='0');
       model outcome = c1 c1*c1 c2 c3;
       bootstrap bootci;
run;


/*Propensity Score Estimation*/
proc logistic data=sim;
       model exposure(event='1') = c1 c1*c1 c2 c3 c2*c3 ;
       output out=sim_ps p = pshat;
run;


/*PS Method 1: Propensity Score Stratification*/
proc psmatch data=sim region=allobs;
       class exposure;
       psmodel exposure(treated='1') = c1 c1*c1 c2 c3 c2*c3;
       strata nstrata=10 key=total;
       assess ps var=(outcome) / varinfo;
run;


/*PS Method 2: Propensity Score Regression Adjustment*/
proc glm data=sim_ps;
       model outcome = exposure pshat / solution;
run; quit;
```

```
/*PS Method 3: Propensity Score Matching*/
proc psmatch data=sim;
        class exposure;
        psmodel exposure(treated='1') = c1 c1*c1 c2 c3 c2*c3;
        match distance = lps method = greedy (k=1) caliper = 0.2;
        assess ps var=(c1 c2 c3 outcome) / varinfo stddev = pooled ;
        output out(obs=match) = sim_matched matchid = _MatchID;
run;
proc psmatch data=sim;
        class exposure;
        psmodel exposure(treated='1') = c1 c2 c3;
        match distance = lps method = greedy (k=1) caliper = 0.2;
        assess ps var=(c1 c2 c3 outcome) / varinfo stddev = pooled ;
        output out(obs=match) = sim_matched matchid = _MatchID;
run;


/*PS Method 4: Inverse Probability Weighting*/
proc causaltrt data=sim method = ipw covdiffps;
        class exposure c2 c3;
        psmodel exposure(event='1') = c1 c1*c1 c2 c3 c2*c3 / plots=(psdist
pscovden);
        outcome outcome;
        bootstrap bootci;
run;
proc causaltrt data=sim method = ipw covdiffps;
        class exposure c2 c3;
        psmodel exposure(event='1') = c1 c2 c3 / plots=(psdist pscovden);
        outcome outcome;
        bootstrap bootci;
run;
```

eMaterials 3. Overview of propensity score stratification and regression adjustment

Stratification

Stratification involves grouping subjects into strata with similar values of estimated PS (e.g., quintiles).[15] The simple comparison of the average outcomes among the exposed vs. the unexposed within each PS stratum,S_{ps}, estimates the stratum-specific conditional effects:

$$E[Y^{a=1} - Y^{a=0} \mid S_{ps}]$$

Two critical points merit attention. First, the estimated stratum-specific conditional exposure effects may differ in size when effect modification by the PS strata is present. The marginal effect can be estimated as a weighted average of the stratum-specific conditional effect estimates with the weights being the proportions of individuals in each stratum to the entire study sample. Second, the PS stratification assumes conditional exchangeability holds given the PS "strata." That is, the estimated PS values within each stratum need to be "similar enough" so that conditioning on the strata approximates conditioning the PS themselves.[16]

Determining the optimal number of the PS strata requires considering a bias-variance trade-off. If the number of PS strata is too small, PS can be widely distributed within each stratum. Consequently, even within the same PS stratum, the exposed and unexposed individuals may have differential distributions of PS and, hence, observed confounders (bias). In contrast, increasing the number of strata may better adjust for confounding, but using too many strata can result in small stratum-specific sample sizes and imprecise estimates (large variance).[16]

Regression adjustment

The estimated PS can also be used as a single adjustment variable in the outcome regression analysis.[17,18] For example, one may specify the following outcome model:

$$E[Y|A, PS] = \gamma_0 + \gamma_1 A \ + \gamma_2 PS$$

In this model, $\gamma_1$corresponds to $E[Y|A = 1, PS] - E[Y|A = 0, PS]$, which is the conditional effect $E[Y^{a=1} - Y^{a=0} \mid PS]$ (i.e., the exposure effect among individuals with identical PS) under the identifiability assumptions.

There are two distinctions worth highlighting between PS regression adjustment and PS stratification. First, PS regression adjustment estimates conditional outcome expectations given PS themselves while the PS stratification estimates conditional outcome expectations given PS "strata." Thus, regression adjustment is not prone to residual confounding bias that crude stratification may suffer. Second, while PS stratification only involves propensity model specification, the regression adjustment approach also specifies the outcome model conditional on PS. Thus, PS regression adjustment makes additional modeling assumptions (e.g., linearity for PS and effect homogeneity by omitting product terms between A and PS).

eMaterials 4. Comparison of propensity score stratification, regression adjustment, matching, and inverse probability weighting

In the main text, we focused on PSM and IPW. We provide comparisons with those methods with other two PS-based methods (i.e., stratification and regression adjustment). The discussion points are summarized in **eTable 1**.

1. The PS methods rely on the same assumptions for exchangeability and consistency but deal with the positivity assumption differently

The four PS methods all rely on the same identifiability assumptions of conditional exchangeability and consistency. In contrast, the PS methods all assume positivity conditional on PS but take different approaches to handle potential positivity violations. PS stratification makes positivity violations conditional on the PS "strata" explicit by assessing whether each PS stratum contains observations from both exposed and unexposed individuals. Positivity given the PS strata allows non-parametric estimation of causal effects after PS estimation even if positivity violations given PS does not strictly hold. However, this feature of PS stratification comes at cost of potential residual confounding due to crude PS strata. Regression adjustment uses model extrapolation in estimating conditional expectations although the positivity violations can at least partly be mitigated by trimming observations with extreme PS values. In IPW, individuals will receive substantially large or small weights when their covariate patterns potentially violate positivity. Trimming such observations with extreme weights is often recommended.[19] Lastly, PSM explicitly addresses potential positivity violations by excluding those who have extreme PS values and, thus, cannot be matched (so-called "off-support" individuals). While such explicit handling of positivity violations is the advantage of the PS methods, one caveat is that causal

estimand of interest generally changes after excluding individuals who potentially violate positivity.[20]

2. Although the PS methods make the same exchangeability assumption, some methods suffer from residual confounding

The four PS methods are all based on the same conditional exchangeability (i.e., no confounding conditional on measured covariates). However, PS stratification does not fully eliminate bias by the measured covariates, especially when strata are crude. Similarly, PSM may result in an insufficient balance of the measured covariates when the pre-specified caliper is wide. On the other hand, PS regression adjustment and IPW do not suffer from residual confounding, assuming the models involved are correctly specified.

3. The PS methods make different modeling assumptions after propensity score estimation

All PS methods specify a propensity model to estimate PS. Stratification and PSM often do not require any further modeling once PS is estimated. In contrast, regression adjustment specifies an outcome model conditional on PS and makes extra modeling assumptions (e.g., linearity for PS). IPW also specifies a weighted outcome model to approximate a marginal structural model, but the outcome model tends to make fewer assumptions than it does in PS regression adjustment or even be saturated (no modeling assumption) when estimating the marginal effect of a single-point binary exposure.

4. The PS methods target different causal estimands (i.e., each method answers a different research question)

The alternative PS methods often target different causal estimands.[20–22] In other words, when an effect estimate from one PS method differs from an estimate from another PS method, they can both be correct but simply answer different questions. Stratification estimates the PS strata-specific effects, which can be standardized over the PS strata distribution to estimate a marginal effect. Regression adjustment technically estimates conditional effects given PS but often produces a single effect estimate by assuming effect homogeneity (no product term between exposure and PS). Combining PS regression adjustment with standardization can more flexibly estimate a marginal effect but has rarely been done in practice. PSM estimates a marginal effect in a population represented by a matched sample. Because the matched sample excludes individuals with extreme PS values, PSM does not estimate an exposure effect among individuals who would always or never be exposed unless they were intervened and forced to have an alternative exposure level. PSM often uses all exposed individuals and matches them with their unexposed pairs. This approach will estimate an exposure effect among the people who were in fact exposed (i.e., average treatment effect in the treated [ATT]).[23] IPW can estimate both marginal and conditional effects, depending on the definition of weights and specification of a marginal structural model.

eTable 1. Comparison of alternative analytic methods by the underlying assumptions

Analytic Approach	Features		
	Causal Estimand	Identifiability Assumptions	Model Specifications
Multivariable Regression	• Conditional effects within the covariate strata<br>• Marginal effect assuming no effect modification by any of the measured covariates	• Conditional exchangeability based on the covariates used in the PS estimation<br>• Potential residual confounding due to crude strata<br>• Consistency for the exposure of interest<br>• Positivity violation if only exposed or unexposed individuals are present in the PS stratum	• Propensity model for probabilities of being exposed conditional on measured covariates.
PS Stratification	• Conditional effects within the PS strata<br>• Marginal effect as a weighted average of the PS stratum-specific conditional effects	• Conditional exchangeability based on the covariates used in the PS estimation<br>• Potential residual confounding due to crude strata<br>• Consistency for the exposure of interest<br>• Positivity violation if only exposed or unexposed individuals are present in the PS stratum	• Propensity model for probabilities of being exposed conditional on measured covariates.
PS Regression Adjustment	• Conditional effects for individuals with an identical PS value<br>• Marginal effect assuming no effect modification by PS.	• Conditional exchangeability based on the covariates used in the PS estimation<br>• Consistency for the exposure of interest<br>• Positivity violation is addressed via model extrapolation with modeling assumptions.	• Propensity model for probabilities of being exposed conditional on measured covariates.<br>• Outcome model conditional on an exposure and estimated PS
PS Matching	• Marginal effect in the population represented by the matched sample, which excludes individuals with extreme PS values from the original sample.	• Conditional exchangeability based on the covariates used in the PS estimation<br>• Potential residual confounding due to wide caliper distance<br>• Consistency for the exposure of interest<br>• Positivity is ensured by excluding unmatched individuals.	• Propensity model for probabilities of being exposed conditional on measured covariates.<br>• Outcome model can be used after matching with caution.
IPW	• Conditional effects by including an additional covariate in the weighted outcome model<br>• Marginal effect in the original population	• Conditional exchangeability based on the covariates used in the PS estimation<br>• Consistency for the exposure of interest<br>• Potential positivity violation is detected as extremely large or small weights, which can be discarded before weighting.	• Propensity model for probabilities of being exposed conditional on measured covariates.<br>• IP-weighted outcome model conditional on exposure (and an additional covariate if estimating conditional effects).

IPW, inverse probability weighting; PS, propensity score.

References for Supplemental Materials

1. Rubin DB. Estimating causal effects of treatments in randomized and nonrandomized studies. *J Educ Psychol*. 1974;66(5):688-701.

2. Petersen ML, van der Laan MJ. Causal models and learning from data: integrating causal modeling and statistical estimation. *Epidemiology*. 2014;25(3):418-426.

3. VanderWeele TJ, Knol MJ. A Tutorial on Interaction. *Epidemiol Method*. 2014;3(1):33-72.

4. Hernán MA, VanderWeele TJ. Compound treatments and transportability of causal inference. *Epidemiology*. 2011;22(3):368-377.

5. Hernán MA, Robins JM. *Causal Inference: What If*. Boca Raton: Chapman & Hall/CRC; 2020.

6. Greenland S, Robins JM. Identifiability, exchangeability, and epidemiological confounding. *Int J Epidemiol*. 1986;15(3):413-419.

7. Greenland S, Robins JM. Identifiability, exchangeability and confounding revisited. *Epidemiol Perspect Innov*. 2009;6:4.

8. Maldonado G, Greenland S. Estimating causal effects. *Int J Epidemiol*. 2002;31(2):422-429.

9. Greenland S, Pearl J, Robins JM. Causal diagrams for epidemiologic research. *Epidemiology*. 1999;10(1):37-48.

10. VanderWeele TJ. Principles of confounder selection. *Eur J Epidemiol*. 2019;34(3):211-219.

11. Hernán MA, Hernández-Díaz S, Werler MM, Mitchell AA. Causal knowledge as a prerequisite for confounding evaluation: an application to birth defects epidemiology. *Am J Epidemiol*. 2002;155(2):176-184.

12. Hernán MA. Does water kill? A call for less casual causal inferences. *Ann Epidemiol*. 2016;26(10):674-680.

13. VanderWeele TJ. Concerning the consistency assumption in causal inference. *Epidemiology*. 2009;20(6):880-883.

14. Westreich D, Cole SR. Invited commentary: positivity in practice. *Am J Epidemiol*. 2010;171(6):674-677; discussion 678-81.

15. Rosenbaum PR, Rubin DB. Reducing Bias in Observational Studies Using Subclassification on the Propensity Score. *J Am Stat Assoc*. 1984;79(387):516-524.

16. Lunceford JK, Davidian M. Stratification and weighting via the propensity score in estimation of causal treatment effects: a comparative study. *Stat Med*. 2004;23(19):2937-2960.

17. Rosenbaum PR, Rubin DB. The central role of the propensity score in observational studies for causal effects. *Biometrika*. 1983;70(1):41-55.

18. Vansteelandt S, Daniel RM. On regression adjustment for the propensity score. *Stat Med*. 2014;33(23):4053-4072.

19. Stürmer T, Rothman KJ, Avorn J, Glynn RJ. Treatment effects in the presence of unmeasured confounding: dealing with observations in the tails of the propensity score distribution--a simulation study. *Am J Epidemiol*. 2010;172(7):843-854.

20. Hill J. Discussion of research using propensity-score matching: Comments on 'A critical appraisal of propensity-score matching in the medical literature between 1996 and 2003'by Peter Austin, Statistics in Medicine. *Stat Med*. 2008;27(12):2055-2061.

21. Lunt M, Solomon D, Rothman K, et al. Different methods of balancing covariates leading to different effect estimates in the presence of effect modification. *Am J Epidemiol*. 2009;169(7):909-917.

22. Kurth T, Walker AM, Glynn RJ, et al. Results of multivariable logistic regression, propensity matching, propensity adjustment, and propensity-based weighting under conditions of nonuniform effect. *Am J Epidemiol*. 2006;163(3):262-270.

23. Austin PC. An Introduction to Propensity Score Methods for Reducing the Effects of Confounding in Observational Studies. *Multivariate Behav Res*. 2011;46(3):399-424.
